# Successful Multidisciplinary Treatment of Severe Pyogenic Liver Abscess Caused by Fish Bone: A Case Report

**DOI:** 10.7759/cureus.71102

**Published:** 2024-10-08

**Authors:** Kei Harada, Takahisa Fujikawa, Yusuke Uemoto, Taisuke Matsuoka, Yuichiro Kawamura

**Affiliations:** 1 Surgery, Kokura Memorial Hospital, Kitakyushu, JPN

**Keywords:** fish bone, foreign body, multidisciplinary treatment, pyogenic liver abscess, sepsis

## Abstract

Pyogenic liver abscess (PLA) is a potentially fatal infection that can lead to sepsis and shock. Among the various causes of PLA, ingestion of foreign bodies such as fish bones is relatively rare. Unless there are specific symptoms such as painful swallowing, patients rarely remember having ingested foreign bodies, making it often difficult to identify the cause of PLA. In addition, the treatment strategy and perspectives for PLA caused by foreign bodies are controversial. Herein, we present a successful case of multidisciplinary treatment for sepsis due to a PLA caused by a fish bone with a literature review.

## Introduction

Pyogenic liver abscess (PLA) is a rare but potentially life-threatening disease with a fatality rate of 6-19% all over the world [1, 2]. The most common etiologies of PLA include complications of cholangitis, hematogenous spread in systemic sepsis, and trauma [3, 4]. However, PLA due to the ingestion of a foreign body such as a fish bone is rare [3, 5]. Unless specific symptoms such as odynophagia are present, patients rarely recall having ingested a foreign body, and they may present with nonspecific symptoms such as loss of appetite, vomiting, and weight loss, making etiological diagnosis difficult in many cases [6, 7]. Therefore, PLA due to foreign body ingestion can lead to severe and potentially fatal conditions if detection and treatment are delayed.

One of the most serious consequences of PLA is sepsis, which can result in multiple organ failure, septic shock, and even death [8, 9]. Timely diagnosis and multidisciplinary treatment are very important to improve the patient's prognosis [1, 4, 10]. Still, the treatment strategy for PLA caused by foreign bodies remains controversial.

Herein, we present a successful case of multidisciplinary treatment for sepsis due to a PLA caused by a fish bone.

## Case presentation

A 69-year-old man sought medical advice at our hospital with the main complaints of fever and epigastric pain, and he was referred to our department for further examination and treatment. Medical history revealed that the patient had been experiencing discomfort in the anterior chest and loss of appetite for the past five days and had been eating poorly. The patient’s past medical history was only significant for hypertension. The vital signs recorded were as follows: blood pressure 91/62 mmHg, pulse rate 102 beats/min, and body temperature 39.2°C. The abdomen was soft, non-distended, but diffusely tender to palpation with most tenderness localized to the upper quadrant region, and bowel sounds were present with no rebound or guarding. Laboratory analysis showed C-reactive protein of 30.1 (reference value: 0.0-0.14) mg/L, white blood cell counts of 45.8 (3.3-8.6) × 10^3^/uL, neutrophils of 92.8%, blood urea nitrogen of 50.3 (8-20) mg/dL, creatinine of 3.96 (0.65-1.0) mg/dL, hemoglobin of 9.5 (13.7-16.8) g/dL, and platelets of 9.6 (15.8-34.8) × 10^3^/μL. The basic metabolic panel was within normal limits and blood glucose was 113 (73-109) mg/dL. Liver enzymes were also elevated with aspartate transaminase of 190 (13-30) U/L, alanine transaminase of 81 (10-30) U/L, and alkaline phosphatase of 445 (38-113) U/L. Coagulation tests showed Prothrombin Time Activity of 41 (70-130) %, Activated Partial Thromboplastin Time of 44.9 (24-39) seconds, D-dimer of 62.9 (<1.0) ng/mL, and Fibrinogen Degradation Products of 42.0 (<5.0) μg/mL. The data are shown in Table 1.

**Table 1:** Laboratory findings. CRP: C-reactive protein, WBC: white blood cell counts, BUN: blood urea nitrogen, AST: aspartate transaminase, ALT: alanine transaminase, ALP: alkaline phosphatase, PT: Prothrombin Time Activity, APTT: Activated Partial Thromboplastin Time, FDP: Fibrinogen Degradation Products

Parameter	Result	Reference Range
CRP	30.1 mg/L	0.0-0.14 mg/L
WBC	45.8 × 10^3^/uL	3.3-8.6 × 10^3^/uL
BUN	50.3 mg/dL	8.0-20.0 × 10^3^/uL
Creatinine	3.96 mg/dL	0.65-1.0 mg/dL
Hemoglobin	9.5 g/dL	13.7-16.8 g/dL
Platelet	9.6 × 10^3^/μL	15.8-34.8 × 10^3^/μL
Glucose	113 mg/dL	73-109 mg/dL
AST	190 U/L	13-30 U/L
ALT	81 U/L	10-30 U/L
ALP	445 U/L	38-113 U/L
PT	41%	70-130%
APTT	44.9 seconds	24-39 seconds
D-dimer	62.9 ng/mL	<1.0 ng/mL
FDP	42.0 μg/mL	<5.0 μg/mL

**Figure 1:**
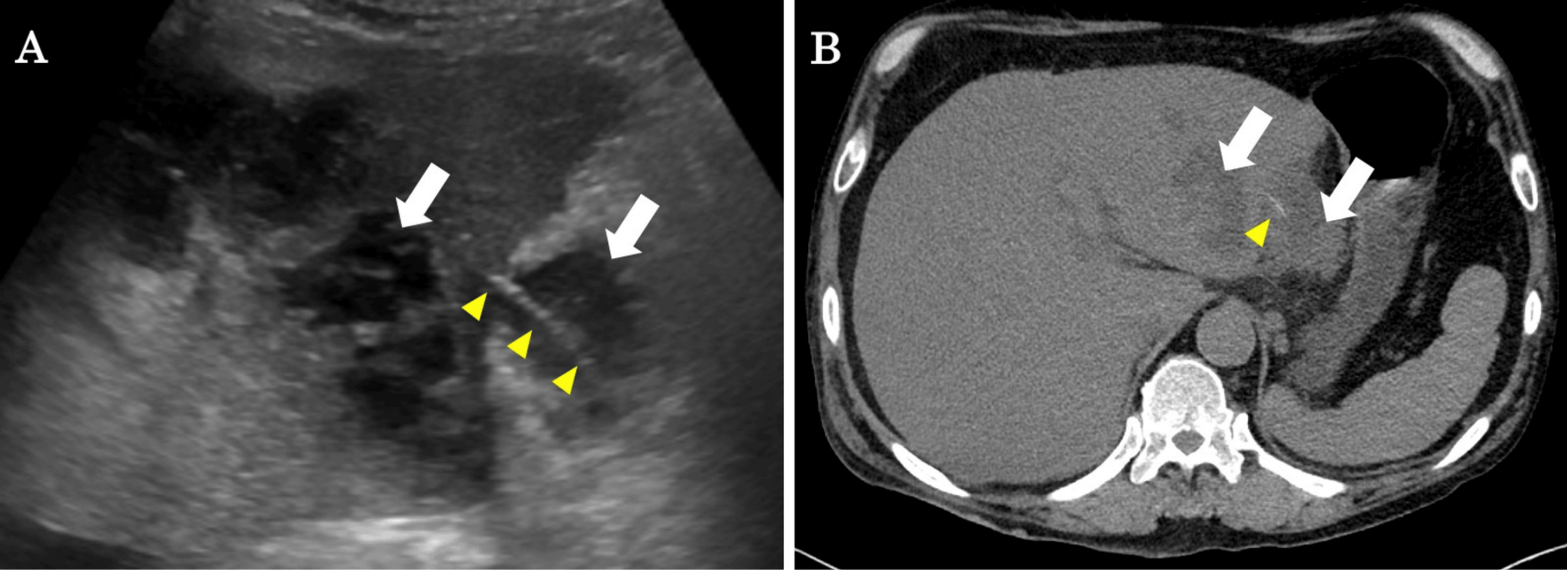
Abdominal ultrasound and CT findings. (A) There are lesions suspected to be abscesses in the left lobe of the liver and adjacent to the liver (white arrows). A linear hyperechoic lesion was observed penetrating from the extrahepatic mass into the liver (yellow arrows). (B) Low attenuation area lesions suspected to be abscesses (white arrows) and linear high attenuation area lesions suspected to be foreign bodies (yellow arrow) were found. CT: computed tomography

Abdominal ultrasound revealed lesions suspected to be abscesses in the left lobe of the liver and adjacent to the liver, as well as a linear hyperechoic lesion penetrating the liver (Figure 1A). We also performed a computed tomography (CT) of the chest, abdomen, and pelvis without contrast because the patient presented with renal dysfunction. CT scans also showed findings suggestive of abscesses both inside and outside the liver, and a linear shadow was also seen penetrating the liver from the abscess adjacent to the liver (Figure 1B).

**Figure 2:**
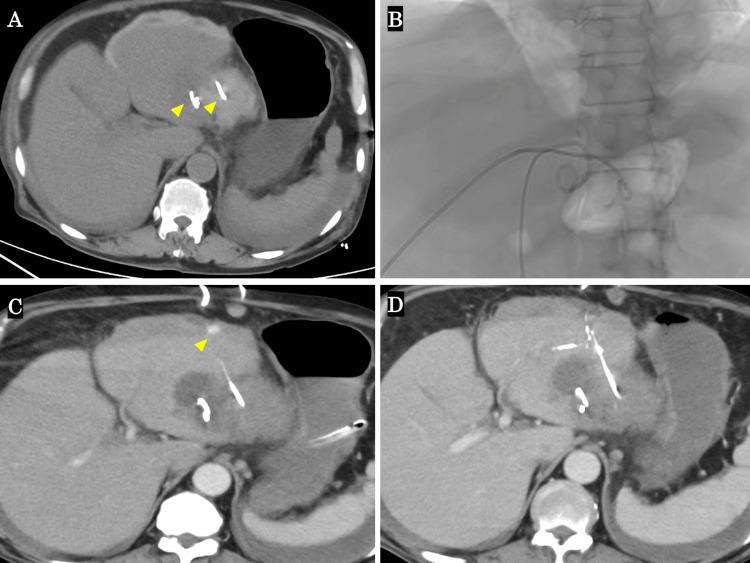
Plain CT and contrast-enhanced CT findings. (A, B) Liver abscess drainage was performed using a combination of ultrasound guidance and fluoroscopy (yellow arrows). (C) Extravasation of the contrast medium was observed on the surface of the liver (yellow arrow). (D) TAE was performed on the hepatic artery A3. CT: computed tomography, TAE: transcatheter arterial embolization

Based on these data, the clinical findings, and the patient's dietary history of having eaten horse mackerel seven days ago, we diagnosed the condition as a suspected liver abscess caused by gastrointestinal penetration due to an ingested fish bone. The patient was suffering from sepsis and acute disseminated intravascular coagulation (DIC) due to a severe liver abscess, so he was admitted to the hospital with the aim of first stabilizing his overall condition and then undergoing surgery to remove the foreign body after his condition improved [11]. From the day of admission, the patient was administered a carbapenem antibiotic for infection and a recombinant human thrombomodulin for acute DIC. The day after admission, percutaneous drainage was performed using ultrasound guidance combined with fluoroscopy (Figure 2A-2B). After drainage, the patient's respiratory condition rapidly deteriorated, so we performed tracheal intubation to stabilize his general condition. Fortunately, the patient was successfully extubated the next day, and his vital signs were maintained without the use of catecholamines. Over the next few days, the inflammatory response steadily decreased and vital signs remained stable, but on the sixth day after admission, the patient suddenly experienced abdominal distension and a sudden drop in Hb levels. A contrast CT scan showed extravasation of contrast medium on the surface of the liver at the puncture route where abscess drainage was performed (Figure 2C). Hepatic artery injury due to abscess drainage was suspected, and emergency interventional radiology (IVR) was performed. Since bleeding was observed from the A3 hepatic artery, transcatheter arterial embolization (TAE) was performed (Figure 2D).

**Figure 3:**
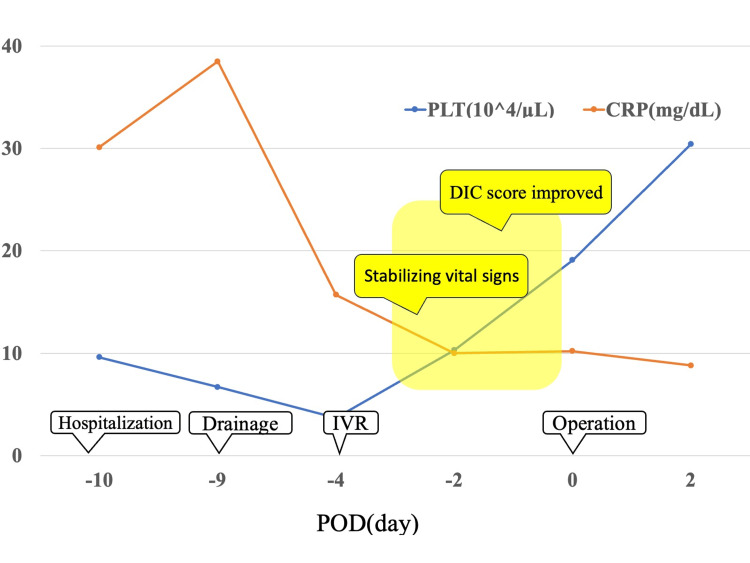
Clinical course of the patient. The figure is the authors’ own creation. PLT: platelet, CRP: C-reactive protein, DIC: disseminated intravascular coagulation, IVR: interventional radiology, POD: postoperative day

As a result of prompt treatment, there was no deterioration in vital signs. The patient was subsequently started on a diet and continued with conservative treatment. However, although the inflammatory response had improved since admission, it was still slightly elevated, and the liver abscess remained. On the 10th day after admission, the DIC score had improved and vital signs had stabilized, so it was determined that the patient had good surgical tolerance, and it was decided to perform surgery to remove the foreign body and drain the abscess (Figure 3).

**Figure 4:**
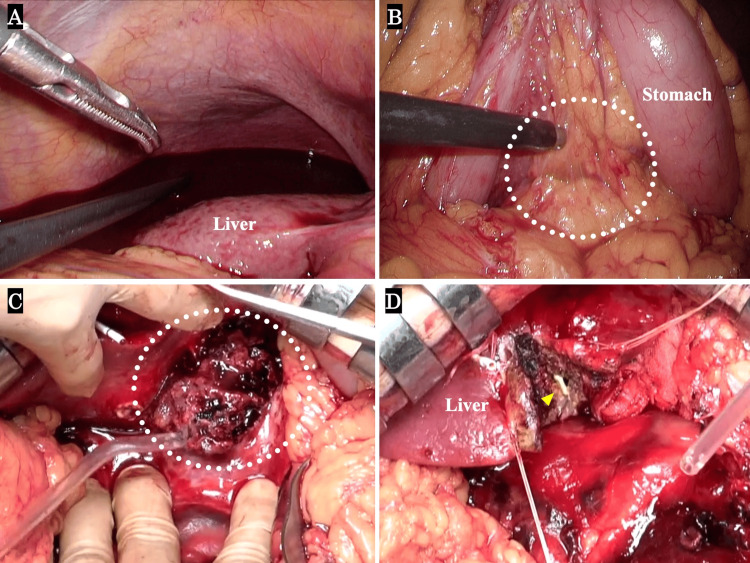
Intra-abdominal findings during operation. (A) Bloody ascites was found to be present under the right diaphragm and around the surface of the liver. (B) The extrahepatic abscess area, which could be confirmed through the omental bursa (white dotted areas), was tightly adhered and difficult to dissect. (C) Extrahepatic abscess containing a fish bone (white dotted areas). (D) Liver abscess curettage and drainage were performed, and the drainage tube (yellow arrow) was visible.

The surgical procedures and findings are shown. The surgery was started under general anesthesia. First, the abdominal cavity was observed with a laparoscope. Bloody ascites was found to be present under the right diaphragm and around the surface of the liver (Figure 4A). We attempted to open the omental bursa and approach the extrahepatic abscess site, but it had become strongly adherent and was difficult to dissect (Figure 4B). Further dissection was difficult using laparoscopy, so open surgery was performed. By continuing the dissection along the liver toward the lesser curvature of the stomach, an extrahepatic abscess was discovered (Figure 4C). We also performed curettage and drainage of the intrahepatic abscess (Figure 4D).

**Figure 5:**
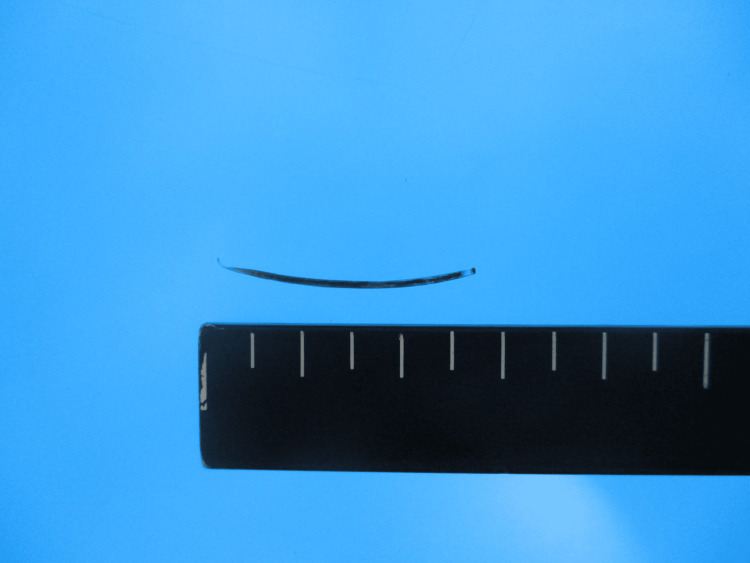
A fish bone from a horse mackerel. A foreign body that appeared to be a fish bone from a horse mackerel, 2.5 cm in size, was found within the abscess.

When the extrahepatic abscess was removed, a foreign body that appeared to be a fish bone, 2.5 cm in size, was found within the abscess (Figure 5). We confirmed using an ultrasound that there was no residual abscess and then completed the surgery. *Streptococcus constellatus* was isolated from the abscess that was removed. Therefore, we diagnosed this clinical course as pyogenic liver abscess (PLA) due to digestive tract penetration by an ingested fish bone. The postoperative course was uneventful, and the patient was discharged on postoperative day (POD) 22. In reporting the case, we respected ethical considerations, including the protection of personal privacy, and provided sufficient informed consent to the patient.

## Discussion

A liver abscess is a potentially fatal consequence of infections caused by bacteria, fungi, protozoa, and worms. It is a small, frequently encysted, purulent inflammation with parenchymal necrosis [7, 12]. Among liver abscesses, PLA occurs worldwide and their frequency is increasing. The incidence of PLA in the East is up to 17.59 per 100,000 people, while the incidence in the West is 1.07 to 3.59 per 100,000 people [13]. The mortality rate from PLA has been reported to be 6-19%, and factors that increase the risk of death include older age, sepsis, shock, acute respiratory distress syndrome, DIC, immunocompromised states, diabetes, and tumors [1, 2, 14].

PLA can develop when bacteria spread hematogenously from a severe septic process, via the portal vein and then via the biliary tract, or due to the spread of an inflammatory process to the liver from adjacent areas, trauma, or after surgery [7, 12]. The incidence of gastrointestinal foreign body perforation has been reported to be less than 1%, and the majority of ingested foreign bodies (80% to 90%) naturally pass through the gastrointestinal tract within a week [5]. Therefore, PLA caused by the invasion of a foreign body (fish bone) into the digestive tract, as in our case, is rare.

Empirical broad-spectrum parenteral antibiotic therapy remains one of the basic treatments for PLA. In most patients, in addition to antibiotics, ultrasound- or CT-guided percutaneous aspiration or catheter drainage is required. Percutaneous fine-needle aspiration ultrasound- or CT-guided percutaneous fine-needle aspiration (PNA) is also used to treat small abscesses less than 5 cm in size [15]. Due to the development of these treatments, the importance of surgical intervention in the treatment of PLA has declined in recent decades. However, there are still indications for it, such as very large (>5 cm) or multilocular abscesses, failure of percutaneous drainage, the presence of intra-abdominal infection (peritonitis), and abscess rupture and, as in our case, liver abscess with foreign body contamination [16].

In this case, in which the patient presented with septic shock due to a severe PLA, we performed conservative treatment with puncture drainage, and then performed surgery after improving the acute DIC and stabilizing vital signs. Although this was a successful case with no obvious perioperative complications, it is unclear whether this strategy was appropriate. Basically, foreign bodies in the abdominal cavity, such as fish bones, should be removed early to prevent organ damage [17]. However, cases of a fish bone penetrating the digestive tract and causing a PLA are rare, and further study is needed to determine treatment strategies and perspectives. Furthermore, it is clear that early diagnosis and treatment are important for PLA caused by foreign bodies, so clinical practice with this in mind would be recommended [1, 4].

## Conclusions

We reported a successful case of severe pyogenic liver abscess caused by a fish bone that developed into sepsis and was treated by multidisciplinary treatment. By prioritizing the treatment for sepsis with stabilization of the patient's general condition and non-surgical drainage of the pyogenic liver abscess, surgery could be performed under favorable conditions. Our case report suggests that timely diagnosis and multidisciplinary treatment are important to improve the prognosis of patients with severe pyogenic liver abscesses. It may also contribute to the consideration of treatment strategies for rare cases such as ours.
